# Subchronic toxicity of oral deltamethrin in laying chickens

**DOI:** 10.3389/fvets.2022.1079580

**Published:** 2022-12-07

**Authors:** Yiming Liu, Mingyue Han, Chunshuang Liu, Yaoxin Tang, Ming Jia, Xiaojie Chen, Haijun Liang, Yunfeng Gao, Xu Gu

**Affiliations:** ^1^Key Laboratory of Animal Antimicrobial Resistance Surveillance, Ministry of Agriculture and Rural Affairs, Feed Research Institute, Chinese Academy of Agricultural Sciences, Beijing, China; ^2^Laboratory of Quality and Safety Risk Assessment for Products on Feed-Origin Risk Factor Ministry of Agriculture and Rural Affairs, Feed Research Institute, Chinese Academy of Agricultural Sciences, Beijing, China; ^3^China Feed Industry Association, Beijing, China; ^4^Heilingjiang Technical Appraisal Station of Agricultural Products, Veterinary Pharmaceuticals and Feed Harbin, Harbin, China

**Keywords:** deltamethrin, laying chickens, subchronic toxicity, relative organ weight, blood biochemical indexes, pathology

## Abstract

Pyrethroid pesticides, with low toxicity to birds and mammals and short persistence in the environment, are widely used now. With the development of intensive poultry farming, pesticide application leads to residues in poultry products and pollution in ecological environment. The aim of the present study was to examine deltamethrin subchronic toxicity in laying chickens. One hundred and twelve laying chickens were randomly assigned to 14 groups including 13 groups medicated with deltamethrin (*n* = 8) and one unmedicated group used as control (*n* = 8). Tissue samples were collected during and after administration for weighing and histopathological analysis. A single dose of deltamethrin (20 mg·kg^−1^·BW·d) was administered orally to laying chickens for 14 days. The results showed that deltamethrin has no significant effect on the relative organ weight of laying chickens (*p* > 0.05). The activities of aspartate aminotransferase and cholinesterase in the plasma gradually decreased over time in the medicated group (*p* < 0.05). Plasma concentrations of urea nitrogen, uric acid, cholesterol, triglycerides, and creatinine significantly increased during treatment (*p* < 0.05), and significant liver damage and loss of intestinal villous epithelium were observed. The intestinal wall thickness, villus height, and crypt depth of laying chickens were altered by deltamethrin treatment. During treatment was withdrawn, the intestinal repair was more extensive than the liver repair.

## Introduction

Food safety is an important issue that affects individuals' livelihoods. To ensure food safety, some countries have issued regulations to restrict and ban highly toxic pesticides, demonstrating the high concern placed by governments on food safety issues. With the development of intensive poultry farming, pesticides are playing an important role in animal disease prevention and control. However, pesticide application leads to residues in poultry products and pollution in ecological environment. The application of some insecticides, such as organophosphate, organochlorine, and carbamate insecticides, has declined or been replaced owning to their toxicity on humans and animals (1). Pyrethroid pesticides, with low toxicity to birds and mammals and short persistence in the environment, are widely used now (2).

**Figure 1:**
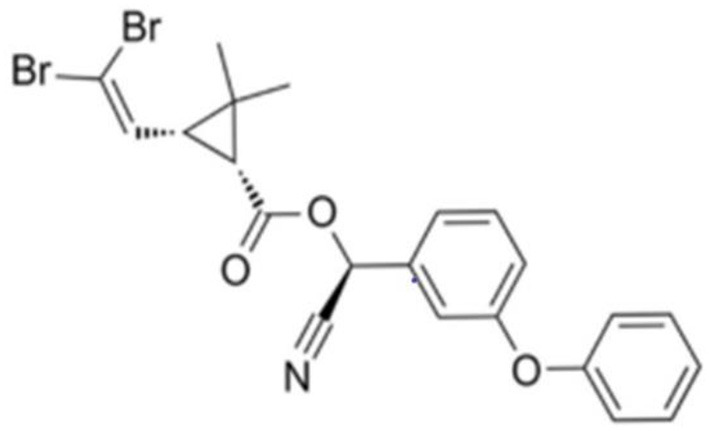
Chemical structure of deltamethrin.

Deltamethrin, one of the most common type II synthetic pyrethroid insecticides, was first marketed in 1977 (3). Deltamethrin is commonly used in agriculture production due to its high efficiency in pest prevention (4), parasite removal (5), and sea lice infection control (5). The chemical structure of deltamethrin is (*S*)-alpha-cyano-3-phenoxybenzyl (1*R*, 3*R*)-3-(2,2-dibromovinyl)-2,2-dimethyl-cyclopropan-1-carboxylate and it has the chemical formula C_22_H_19_Br_2_NO_3_ (Figure 1) (6). One of the metabolites from deltamethrin, 3-(2,2-dibromol)-2,2-dimethyl-(1-cyclopropane) carboxylic acid, has been detected in urine from healthy human population in various countries, although at lower levels than 3-phenoxybenzoic acid (3-PBA), a common metabolite of many pyrethroids (including deltamethrin (7). The presence of unmetabolized deltamethrin has also been reported in human breast milk (8, 9). Deltamethrin is a typical pyrethroid insecticide; it acts on Na^+^ channels to cause excessive depolarisation of the membrane potential, dysmotility or salivation of the insect, and ultimately paralysis and death.

Deltamethrin can be used by the routes of spray or impregnation to protect animals from external parasites (10). However, its widespread application can indirectly endanger the health of livestock and poultry. Direct or indirect exposure to deltamethrin may lead to residual deltamethrin at the level of 0.41–40 U·L^−1^ in human blood (11). Deltamethrin could cause developmental disorders (12), fibrosis (13), and cognitive disorders (14). Due to its high utilization frequency and long half-life, the toxic effects of deltamethrin on the environment, animals, and even humans had been reported (15). In poultry farms, Deltamethrin is commonly used for deworming and parasite control in breeding environments (16–19). Laying chickens may be directly exposed to deltamethrin through the consumption of deltamethrin-contaminated feed and drinking water. In the present study, after orally administration of deltamethrin to laying chickens, we investigated the changes in relative organ weight, biochemical indexes in the blood, and pathological examination of liver and intestinal tissue, aiming to accurately evaluate the subchronic toxic effects of deltamethrin on laying chickens, and to provide a scientific basis for rational use of deltamethrin pesticide.

## Materials and methods

### Chemicals

Deltamethrin standard (99.5% purity) was purchased from Dr. Ehrenstorfer (Augsburg, Germany). Analytical-grade ethanol, anhydrous ethanol, and xylene were purchased from Sinopharm Chemical Reagent Co (Shanghai, China). Total protein (TP) (A045-2), albumin (ALB) (A028-2), aspartate aminotransferase (AST) (C010-2), alanine aminotransferase (ALT) (C009-2), triglyceride (TG) (A110-2), total cholesterol (TC) (A111-2), alkaline phosphatase (ALP) (A059-2), cholinesterase (CHE) (A023-2), urea nitrogen (BUN) (C013-1), uric acid (UA) (C012-2), creatinine (Cr) (C011-2), and lactate dehydrogenase (LDH) (A020-2) were purchased from Nanjing Jian Cheng Bioengineering Institute (Nanjing, China). Harris Hematoxylin solution, 95% eosin, and paraformaldehyde were purchased from Beijing Baiolaibo Technology Co., LTD (Beijing, China). The chemical raw material deltamethrin (98%) was obtained from Hubei Maoerwo Biological Medicine Co., LTD (Hubei, China).

### Experimental design

One hundred and twelve laying chickens were randomly assigned to 14 groups including 13 groups treated with deltamethrin (*n* = 8) and one unmedicated group used as control (*n* = 8). The trial was divided into a deltamethrin treatment period (14 days) followed by a withdrawal period (21 days) during which no deltamethrin was administered. A single dose of deltamethrin (20 mg·kg^−1^·BW·d) was administered orally to laying chickens for 14 days. Food was removed for the 12 h prior to each treatment. All deltamethrin dosing (20mg·kg^−1^·BW·d) was via oral gavage, employing a corn oil vehicle of 1 mL·kg^−1^ body mass. Laying chickens in control group received 1 mL·kg^−1^ corn oil only. One hundred and twelve 52-week-old laying chickens (1.0 ± 0.05 kg) were purchased from Nankou Pilot Base, Chinese Academy of Agricultural Sciences.

### Relative organ weight measurement

Each chicken was weighed at the 1st, 3rd, 7th, 10th, and 14th day during deltamethrin treatment and on the 1st, 3rd, 7th, 10th, 14th, 17th, and 21th day of withdrawal period. Fresh tissues were then collected after slaughter and then weighed: the cockscomb, crop, muscle stomach, glandular stomach, liver, heart, lung, kidney, spleen, and brain. Relative organ weight was calculated using the following formula:


(1) $$\begin{array}{l} \begin{array}{lll} \text{Relative organ weight }(\%)\; & = & \text{ Wet organ weight}/\text{body weight} \end{array}\\ \;\begin{array}{ll} \times & 100\% \end{array} \end{array}$$

### Determination of plasma biochemical indices of laying chickens

At the same time, fresh blood was collected in 15 mL tubes (containing 0.1% heparin sodium solution), then centrifuged at 3,108 × g and 4°C for 10 min. Plasma was collected and stored at −40°C. Plasma was analyzed using an enzyme-linked immunosorbent assay (ELISA) instrument. We measured the concentrations of TP, ALB, globulin (GLB), TG, TC, BUN, UA, and Cr, and the enzyme activities of glutathione transaminase (GOT), AST, glutamate transaminase (GPT), ALT, ALP, CHE, and LDH.

### Histopathology and morphological measurements

Eight laying chickens were selected at random to be sacrificed at six time points (1st, 7th, and 14th day during deltamethrin treatment and 1st, 7th, and 14th day during withdrawal period). For each individual, a 0.5 × 0.5 × 0.5 cm section of liver tissue was collected; intestinal tissue was folded in half and a 1-cm section in the middle was taken and placed in 0.9% sodium chloride to remove residual force. Liver and intestinal tissues were immediately fixed in 4% paraformaldehyde. Fixed liver and intestinal samples were then immersed in 95% ethanol for 6 h; anhydrous ethanol (for 1.5 h, then 1 h); and 1:1 xylene: anhydrous ethanol for 20 min. Both tissues were then immersed in xylene twice; 10 min each time for liver tissue, and 15 min followed by 10 min for intestinal tissue. Tissues were next immersed in soft wax at 50–52°C for 30 min and 1 h, in hard wax at 58–60°C for 1 h. Embedding was carried out at a temperature lower than 65°C, then sections were trimmed. Wax blocks were sliced continuously into 4 μm sections and incubated on a 55°C baking table for 3 h, then stored.

Baked sections were immersed in xylene (five times for 10 min each), anhydrous ethanol (twice for 5 min each); 95% ethanol (twice for 5 min each); 80% ethanol for 5 min; 70% ethanol for 5 min; and deionized water for 3–5 min. Samples were then stained by immersing in Harris haematoxylin solution for 5–10 min; washing in water; color-separating with 0.5% hydrochloric acid alcohol for 3–10 s; bluing for 15–30 min; immersing in 70% ethanol for 5 min, and immersing in 80% ethanol for 5 min. Samples were counterstained by immersing in eosin solution (95% ethanol solution) for 30 s; 95% ethanol (for 1 min, then 5 min); anhydrous ethanol (for 1 min, then for 5 min); 1:1 xylene: anhydrous ethanol for 5 min; then xylene (for 1 min, 5 min, then 5 min). Liver tissue sections were sealed with neutral gum for pathological observation under a microscope. Intestinal tissue sections were observed under a microscope. Three typical locations were selected for each sample, the villus height (V), crypt depth (C) and intestinal wall thickness (D) were measured with an Image Pro-plus 7.0 (Media Cybernetics, USA).

### Data processing

SPSS 20.0 was used to analyze the data using one-way analysis of variance (ANOVA). Duncan's multiple comparison method was used to determine statistically significant differences between the mean values of each group. Significant differences were defined at *p* < 0.05.

## Results and discussion

### Effects of deltamethrin on relative organ weight of laying chickens

The widespread use of deltamethrin increases the risk of toxicity in humans. Some previous studies reported side effects of deltamethrin (20–24). The present study mainly evaluated the toxicity of deltamethrin in laying chickens. Organ weight is often considered an important parameter in toxicological research (25). The effects of daily deltamethrin treatment on relative organ weight in laying chickens are shown in Table 1.

**Table 1:** Effects of deltamethrin on relative organ weight in laying chickens.

**Time (day)**	**Cockscomb**	**Crop**	**Muscular stomach**	**Glandular stomach**	**Liver**	**Heart**	**Lungs**	**Kidney**	**Spleen**
**Drugs administration period**									
0 d	0.41 ± 0.15	0.41 ± 0.15^ab^	2.13 ± 1.94^b^	0.37 ± 0.08^ab^	2.71 ± 0.70^b^	0.43 ± 0.03^ab^	0.46 ± 0.09^cd^	0.63 ± 0.15^bcd^	0.11 ± 0.03^ab^
1 d	0.69 ± 0.15	0.46 ± 0.09^b^	1.47 ± 0.16^ab^	0.41 ± 0.18^b^	1.41 ± 0.89^a^	0.42 ± 0.06^ab^	0.41 ± 0.08^abcd^	0.50 ± 0.10^b^	0.12 ± 0.02^ab^
3 d	0.42 ± 0.11	0.42 ± 0.11^ab^	1.51 ± 0.22^ab^	0.36 ± 0.07^ab^	2.38 ± 0.71^b^	0.37 ± 0.06^ab^	0.42 ± 0.09^bcd^	0.35 ± 0.09^a^	0.13 ± 0.03^b^
7 d	0.50 ± 0.12	0.50 ± 0.13^c^	1.51 ± 0.29^ab^	0.38 ± 0.09^ab^	2.11 ± 0.25^b^	0.39 ± 0.06^ab^	0.37 ± 0.04^ab^	0.53 ± 0.13^bc^	0.09 ± 0.03^ab^
10 d	0.40 ± 0.06	0.40 ± 0.06^ab^	1.53 ± 0.20^ab^	0.38 ± 0.06^ab^	2.38 ± 0.41^b^	0.44 ± 0.04^ab^	0.48 ± 0.14^d^	0.56 ± 0.13^bcd^	0.12 ± 0.03^ab^
14 d	0.37 ± 0.04	0.37 ± 0.04^ab^	1.60 ± 0.35^ab^	0.37 ± 0.04^ab^	2.63 ± 0.48^b^	0.38 ± 0.04^b^	0.38 ± 0.07^abc^	0.61 ± 0.14^bcd^	0.09 ± 0.02^ab^
**Drugs withdrawal period**									
1 d	0.38 ± 0.08	0.38 ± 0.08^ab^	1.52 ± 0.15^ab^	0.32 ± 0.05^ab^	2.74 ± 0.45^b^	0.41 ± 0.03^ab^	0.36 ± 0.06^ab^	0.62 ± 0.11^bcd^	0.10 ± 0.02^ab^
2 d	0.36 ± 0.05	0.36 ± 0.04^ab^	1.51 ± 0.24^ab^	0.31 ± 0.03^ab^	2.59 ± 0.52^b^	0.37 ± 0.04^a^	0.39 ± 0.04^abc^	0.65 ± 0.04^cd^	0.19 ± 0.14^c^
3 d	0.42 ± 0.05	0.42 ± 0.05^ab^	1.38 ± 0.12^ab^	0.32 ± 0.05^ab^	2.76 ± 0.62^b^	0.40 ± 0.07^ab^	0.38 ± 0.05^abc^	0.70 ± 0.15^d^	0.10 ± 0.02^ab^
7 d	0.39 ± 0.90	0.39 ± 0.09^ab^	1.47 ± 0.19^ab^	0.36 ± 0.08^ab^	2.44 ± 0.45^b^	0.39 ± 0.02^ab^	0.37 ± 0.05^ab^	0.61 ± 0.13^bcd^	0.08 ± 0.02^ab^
10 d	0.40 ± 0.05	0.40 ± 0.05^ab^	1.54 ± 0.13^ab^	0.37 ± 0.04^ab^	2.32 ± 0.25^b^	0.40 ± 0.04^ab^	0.37 ± 0.04^abc^	0.65 ± 0.12^bcd^	0.10 ± 0.02^ab^
14 d	0.39 ± 0.06	0.39 ± 0.06^ab^	1.52 ± 0.21^ab^	0.32 ± 0.07^ab^	2.18 ± 0.25^b^	0.42 ± 0.05^ab^	0.38 ± 0.04^abc^	0.59 ± 0.13^bcd^	0.07 ± 0.01^a^
17 d	0.40 ± 0.05	0.40 ± 0.05^ab^	1.45 ± 0.20^ab^	0.32 ± 0.07^ab^	2.35 ± 0.35^b^	0.41 ± 0.04^ab^	0.39 ± 0.05^abc^	0.56 ± 0.12^bcd^	0.09 ± 0.02^ab^
21 d	0.34 ± 0.04	0.35 ± 0.04^a^	1.36 ± 0.23^a^	0.31 ± 0.06^a^	2.17 ± 0.30^b^	0.41 ± 0.07^ab^	0.36 ± 0.01^ab^	0.57 ± 0.13^bcd^	0.08 ± 0.03^ab^

Superscript letters after mean values indicate statistically significant differences at p < 0.05.

We found that the relative weight of cockscomb, crop, muscular stomach, liver, lung, and kidney from treated with deltamethrin chickens decreased compared with the control group. However, there were no significant differences in the relative weight of the glandular stomach (except at the 1st day of treatment), the spleen (except at the 1st and 2nd day of withdrawal), or the heart (*p* > 0.05). Compared with the control group, we found that the relative weight of cockscomb rose significantly at the 1st and 7th day of treatment; the relative weight of crop rose significantly at the 7th day of treatment; the relative weight of glandular stomach rose significantly at the 1st day of treatment; the relative weight of kidney rose significantly at the 7th day during withdrawal; and the relative weight of spleen rose significantly at the 2nd day during withdrawal (*p* < 0.05). The increases of organ weight at specific time points may indicate a compensatory response to the functional requirements for metabolism and excretion of deltamethrin. The decreases in relative weight of organs such as the cockscomb, crop, muscular stomach, liver, lungs, and kidneys were consistent with the results of previous studies, and indicating the deltamethrin toxicity on organs (26–29).

### Effects of deltamethrin on blood biochemical indexes of laying chickens

The pyrethroid insecticide deltamethrin has previously been reported to have toxic effects (30–32). The proteins of TP, GLB, and ALB are involved in cellular structure and function, nitrogen metabolism, and stress responses (33). Fluid biochemical indexes are the main evaluation index for disease diagnosis and analysis. In this study, blood biochemical indexes were measured in laying chickens treated with deltamethrin (Table 2). Decreases in TP, ALB, and GLB in the plasma are the primary manifestation of liver and kidney damage. There was a significant difference in ALB content (*p* < 0.05) only at the 3rd day of deltamethrin treatment. There was no significant difference in plasma TP content during deltamethrin treatment or withdrawal (*p* > 0.05). In general, the results of this study showed stable levels of TP, ALB, and GLB in the plasma.

**Table 2:** Effects of deltamethrin on blood biochemical indexes of laying chickens.

**Time (day)**	**Total protein (TP)**	**Albumin (ALB)**	**Globulin (GLB)**	**Aspertate aminotransferase (AST)**	**Alanine transaminase (ALT)**	**Triglyceride (TG)**	**Total cholesterol (TC)**
	**(g·L^−1^)**	**(g·L^−1^)**	**(g·L^−1^)**	**(U·L^−1^)**	**(U·L^−1^)**	**(mmol·L^−1^)**	**(mmol·L^−1^)**
**Drugs administration period**							
0 d	42.30 ± 1.86^ab^	22.39 ± 1.36^a^	19.53 ± 2.16	135.17 ± 15.30^a^	42.28 ± 8.47^bcd^	4.94 ± 0.58^a^	2.63 ± 0.78^ad^
1 d	42.36 ± 2.35^ab^	22.06 ± 1.31^a^	22.73 ± 1.67	545.08 ± 50.50^g^	49.63 ± 8.25^ab^	5.63 ± 1.29^ab^	1.56 ± 0.39^ab^
3 d	44.24 ± 3.95^ab^	26.23 ± 2.58^b^	19.01 ± 2.52	439.04 ± 64.84^f^	51.59 ± 9.54^abc^	8.15 ± 2.05^abcd^	6.22 ± 1.19^e^
7 d	45.58 ± 1.49^ab^	23.07 ± 2.66^ab^	19.90 ± 0.93	251.80 ± 63.90^c^	47.08 ± 7.02^a^	8.89 ± 2.71^cd^	2.43 ± 1.17^ab^
10 d	45.61 ± 1.16^b^	22.24 ± 2.21^a^	20.05 ± 2.02	354.98 ± 38.54^e^	48.78 ± 6.54^a^	10.65 ± 2.74^d^	3.89 ± 1.60^d^
14 d	42.04 ± 3.02^b^	22.46 ± 1.67^a^	21.12 ± 1.26	294.00 ± 48.37^d^	45.27 ± 2.78^a^	6.36 ± 1.83^abc^	1.16 ± 0.24^a^
**Drugs withdrawal period**							
1 d	43.63 ± 1.71^ab^	25.23 ± 2.92^ab^	20.97 ± 2.32	132.13 ± 1.85^a^	46.60 ± 6.91^a^	6.49 ± 1.83^abc^	2.59 ± 0.43^ab^
2 d	43.44 ± 2.84^ab^	22.88 ± 1.92^ab^	20.76 ± 3.04	129.40 ± 8.08^a^	54.13 ± 6.01^abc^	5.03 ± 1.40^a^	2.99 ± 0.77^cd^
3 d	44.76 ± 4.42^ab^	23.02 ± 1.96^a^	20.68 ± 2.92	123.61 ± 5.93^a^	54.41 ± 9.64^abc^	5.91 ± 0.37^ab^	2.59 ± 0.58^ab^
7 d	41.48 ± 4.08^ab^	22.35 ± 2.87^a^	20.41 ± 2.66	125.00 ± 8.93^a^	48.16 ± 2.83^a^	5.78 ± 0.78^ab^	2.48 ± 0.21^ab^
10 d	42.23 ± 2.07^ab^	22.96 ± 2.95^a^	20.05 ± 5.53	180.67 ± 21.83^b^	51.93 ± 6.36^abc^	8.28 ± 1.52^bcd^	3.20 ± 1.05^cd^
14 d	42.06 ± 1.57^ab^	22.53 ± 0.93^a^	19.55 ± 1.08	144.90 ± 11.71^ab^	58.82 ± 6.70^bcd^	5.23 ± 1.13^ab^	2.42 ± 0.83^ad^
17 d	42.51 ± 4.22^ab^	23.06 ± 2.47^ab^	19.80 ± 3.61	140.57 ± 22.07^ab^	59.97 ± 4.05^cd^	5.09 ± 0.89^a^	2.43 ± 0.66^ab^
21 d	41.26 ± 3.62^ab^	23.82 ± 0.61^ab^	22.28 ± 2.62	142.14 ± 12.22^ab^	66.62 ± 1.91^d^	4.92 ± 1.69^a^	2.84 ± 0.09^cd^
**Time (day)**	**Alkaline phosphatase (ALP)**	**Cholinesterase (CHE)**	**Urea nitrogen (BUN)**	**Uric acid (UA)**	**Creatinine (Cr)**	**Lactic dehydrogenase (LDH)**
	**(King unit** ·**100mL**^−1^**)**	**(U**·**mL**^−1^**)**	**(mmol**·**L**^−1^**)**	**(mg**·**L**^−1^**)**	**(**μ**mol**·**L**^−1^**)**	**(U**·**L**^−1^**)**
**Drugs administration period**						
0 d	360.79 ± 32.06^a^	710.42 ± 60.72^ab^	0.46 ± 0.06^a^	625.53 ± 157.50^a^	0.04 ± 0.02^bcd^	828.21 ± 64.80^abcd^
1 d	345.21 ± 88.33^a^	3828.39 ± 369.16^f^	0.68 ± 0.11^a^	646.15 ± 144.62^a^	0.03 ± 0.01^abcd^	859.20 ± 74.20^d^
3 d	418.35 ± 232.23^ab^	2098.76 ± 351.01^e^	1.47 ± 0.39^b^	1377.29 ± 349.63^d^	0.05 ± 0.00^de^	876.63 ± 81.58^abcd^
7 d	1423.01 ± 265.10^e^	1862.42 ± 200.75^e^	1.53 ± 0.41^b^	1380.95 ± 204.49^d^	0.06 ± 0.02^e^	920.63 ± 45.57^cd^
10 d	1007.02 ± 185.10^d^	1933.93 ± 285.43^e^	2.23 ± 0.49^c^	1406.59 ± 179.90^d^	0.07 ± 0.02^f^	930.14 ± 106.27^d^
14 d	504.37 ± 109.84^abc^	1516.96 ± 292.63^d^	0.46 ± 0.08^a^	1093.41 ± 368.80^cd^	0.02 ± 0.01^a^	926.79 ± 91.96^abcd^
**Drugs withdrawal period**						
1 d	712.52 ± 119.13^c^	1518.35 ± 274.94^d^	0.72 ± 0.31^a^	1040.00 ± 106.95^bc^	0.05 ± 0.01^de^	909.57 ± 54.96^bcd^
2 d	670.19 ± 22.95^bc^	1046.72 ± 195.09^bc^	0.52 ± 0.05^a^	915.00 ± 242.77^abc^	0.04 ± 0.01^bcd^	837.46 ± 159.49^abcd^
3 d	560.68 ± 113.16^abc^	1042.74 ± 207.31^bc^	0.62 ± 0.16^a^	885.00 ± 182.52^abc^	0.05 ± 0.01^cde^	796.21 ± 104.63^a^
7 d	462.03 ± 165.26^abc^	1170.10 ± 155.33^cd^	0.57 ± 0.05^a^	812.50 ± 203.56^abc^	0.04 ± 0.00^bcd^	794.08 ± 108.49^abcd^
10 d	492.24 ± 31.34^abc^	995.98 ± 177.84^bc^	0.67 ± 0.07^a^	741.67 ± 192.14^abc^	0.04 ± 0.01^bcd^	809.11 ± 127.06^abcd^
14 d	309.51 ± 153.55^a^	853.20 ± 187.36^abc^	1.37 ± 0.46^b^	758.87 ± 185.89^abc^	0.04 ± 0.02^bcd^	754.62 ± 132.89^abc^
17 d	326.07 ± 89.05^a^	964.64 ± 257.95^bc^	0.46 ± 0.19^a^	710.64 ± 305.13^ab^	0.03 ± 0.01^abc^	754.99 ± 87.20^abc^
21 d	346.86 ± 164.62^a^	707.63 ± 101.12^ab^	0.47 ± 0.10^a^	685.11 ± 119.79^ab^	0.03 ± 0.01^ab^	748.88 ± 41.43^ab^

Superscript letters after mean values indicate statistically significant differences at p < 0.05.

Deltamethrin is metabolized in the liver (the main metabolic site of heterologous organisms) by hepatic microsomal enzymes, allowing toxic deltamethrin derivatives to accumulate in the liver (31, 34, 35). The kidney is a major excretory organ (36), and therefore the toxic effects of chemicals can be observed ordinarily in kidney tissues (37). Phytochemical, cytotoxic, hepatoprotective, and antioxidant properties of Delonix regialeaves extract (38). Liver and kidney damage are often indicated by changes in the concentrations of marker metabolites and enzymes such as AST, ALT, ALP, and LDH in the liver, and urea, Cr, and renin in the kidney. We here found that AST concentration in the plasma decreased during deltamethrin treatment; AST concentration was significantly higher in medicated groups than in the control group at the 1st day of treatment (*p* < 0.05). However, there was no significant difference between 14th and 21th day of withdrawal (*p* > 0.05), similar to the results of previous studies (39). ALT concentration in the plasma gradually increased over the withdrawal period, with significantly higher content concentration at 21th day of withdrawal than at all other time-points (aside from 0, 14th, and 17th day) (*p* < 0.05). It is observed a significantly higher content of ALP at the 7th day compared to all other time points (*p* < 0.05). LDH concentration was also significantly increased during the first 10 days of deltamethrin treatment. In summary, deltamethrin significantly increased the content of AST, ALT, ALP, and LDH in the serum of laying chickens, consistent with the findings of previous studies (40). The harmful effects of deltamethrin on hepatocyte membranes may have led to liver dysfunction and a subsequent increase in these blood biochemical indicators (41). Hepatocyte necrosis can also lead to enzyme leakage into the blood, increasing the concentration of serum LDH (42).

AST and ALT are widely distributed within the mitochondria of animal tissue cells; they are less active in the plasma under normal circumstances, but when organs damage occurred, such as the heart and liver, their concentration in organs reduced while increased in blood. Recent studies have found significant increases of ALT in plasma in quail treated with other drugs, indicating that ALT cannot be used as a marker for long-term use of deltamethrin specifically (42, 43). ALP is commonly found in the liver, biliary tract, small intestine, bone, lung, and kidney; its elevated levels in the blood indicate damage to the liver or other organs. ALP and LDH are widely distributed in various tissues and organs, and play important roles in regulating and functioning of animal metabolism. In the present experiment, liver cell membrane might be damaged due to deltamethrin treatment, and caused large changes in ALP than LDH levels at the 7th day of treatment. Over the treatment period, ALP in plasma concentration firstly increased and then decreased; during the withdrawal period, it gradually returned to normal levels.

Over the treatment period, CHE in plasma showed a significant but gradual decrease, peaking at the 1st day of treatment at a significantly higher level than all other time points (*p* < 0.05). Deltamethrin was found to induce neurotoxicity by reducing CHE content concentration, consistent with previous findings (44). At the 3rd and 10th day of treatment, the content of TC and TG in plasma, were significantly higher than all other time points (*p* < 0.05). Owning to the lipophilicity of deltamethrin, the liver might absorb a high proportion of deltamethrin, which can induce several liver diseases. These results were generally comparable to those of other studies (39). At the 10th day of treatment, the contents of BUN and Cr in the plasma were significantly higher than at other time points (*p* < 0.05). The same trends was found for uric acid in plasma, except that there was no significant difference between the levels at the 3rd and seventh day during treatment (*p* > 0.05). Increased levels of these biomarkers reflected renal insufficiency. Similar results were obtained in previous studies (41, 45). In summary, we found that daily administration of deltamethrin had significantly increased the blood biochemical indexes of laying chickens, and plasma biochemical indexes gradually decreased to normal levels during deltamethrin treatment was stopped.

### Effects of deltamethrin on histopathology and morphology

Histopathology is a powerful and useful method for assessing the toxic effects of environmental stressors and toxicants, and helpful for visualizing ecotoxicological biomarkers of tissues such as the liver, brain, intestine, and kidney (46). Histopathological changes can reflect the transformation of healthy to unhealthy biological tissues, is more intuitive than single biochemical reactions for assessing the status of an organism.

### Effects of deltamethrin on liver pathology

**Figure 2:**
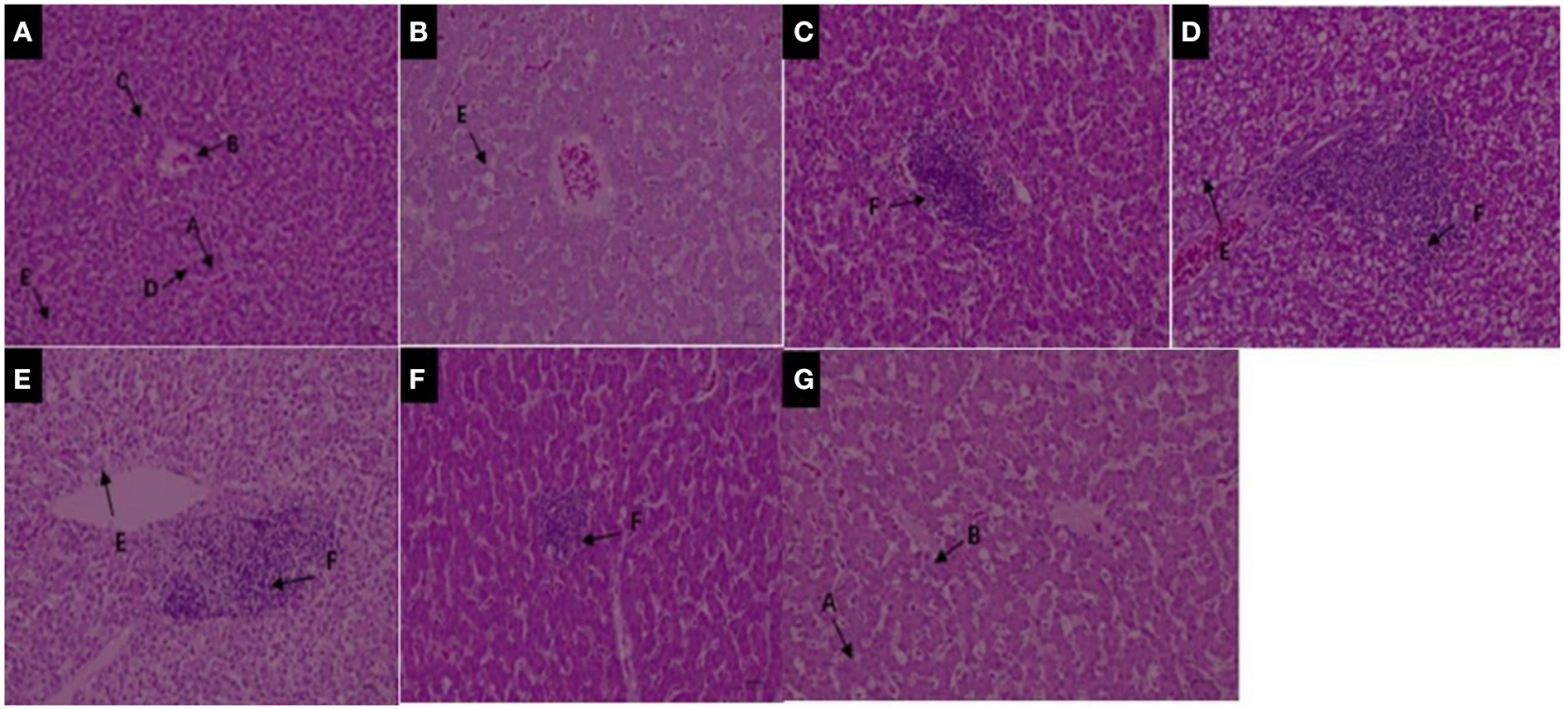
**(A–D)** Liver structure under 200× magnification during deltamethrin treatment for 0 **(A)**, the 1st **(B)**, 7th **(C)**, and 14th day **(D)**. **(E–G)** Liver structure under 200× magnification at the 1st **(E)**, 7th **(F)**, and 14th day **(G)** during deltamethrin treatment withdrawal. A, Hepatic sinusoidal lacunae; B, hepatic veins; C, hepatocytes (cords); D, bile ducts; E, lipid droplet enrichment; F, local lymphocytic infiltration.

Deltamethrin can induce cell damage through activation of multiple pathways. Many studies have shown that deltamethrin exposure can affect cell survival and induce caspase-dependent or -independent apoptosis (47, 48) in the kidney (32), liver (49), neurons (50), and spleen (51). The liver is an important organ for substance metabolism, is involved in the decomposition, synthesis, transformation, storage, phagocytosis, detoxification, and inactivation of various compounds. The liver is therefore a sensitive target of many harmful toxicants, its morphology and physiological and biochemical characteristics are subject to toxin-induced changes. Some studies have shown that pyrethroids cause changes in liver tissue structure, such as tissue bleeding, hepatic sinusoidal dilatation, hepatocyte swelling, water-like degeneration, and inflammatory cell infiltration (52, 53). In this study, we found that hepatocytes were structurally clear and intact at day zero of deltamethrin treatment; they had uniform nucleus size, obvious nucleoli, and clear interphase arrangement of hepatocyte cords and hepatic blood sinusoids, although there was a small amount of local lipid droplet enrichment (Figure 2A). As the treatment time progressed, the impact of deltamethrin on liver tissue structures was observed. Hepatic blood sinusoids were severely damaged, hepatocyte cords were disorganized, numerous lymphocytes were infiltrated locally and around the confluent area, and hepatocyte necrosis appeared in local liver lobules (Figures 2B–D). Compared to day zero of deltamethrin treatment, hepatocytes were significantly more enriched in lipid droplets at the 14th day (Figures 2A,D). During treatment withdrawal, liver tissues did not return to normal, compared with the treatment period, only lipid droplet enrichment decreasing during the withdrawal period (Figures 2E–G); this was comparable to the results of previous studies (33, 54–56). These results indicate deltamethrin has a damage impacts on the liver.

### Effects of deltamethrin on intestinal pathology and morphology

**Figure 3:**
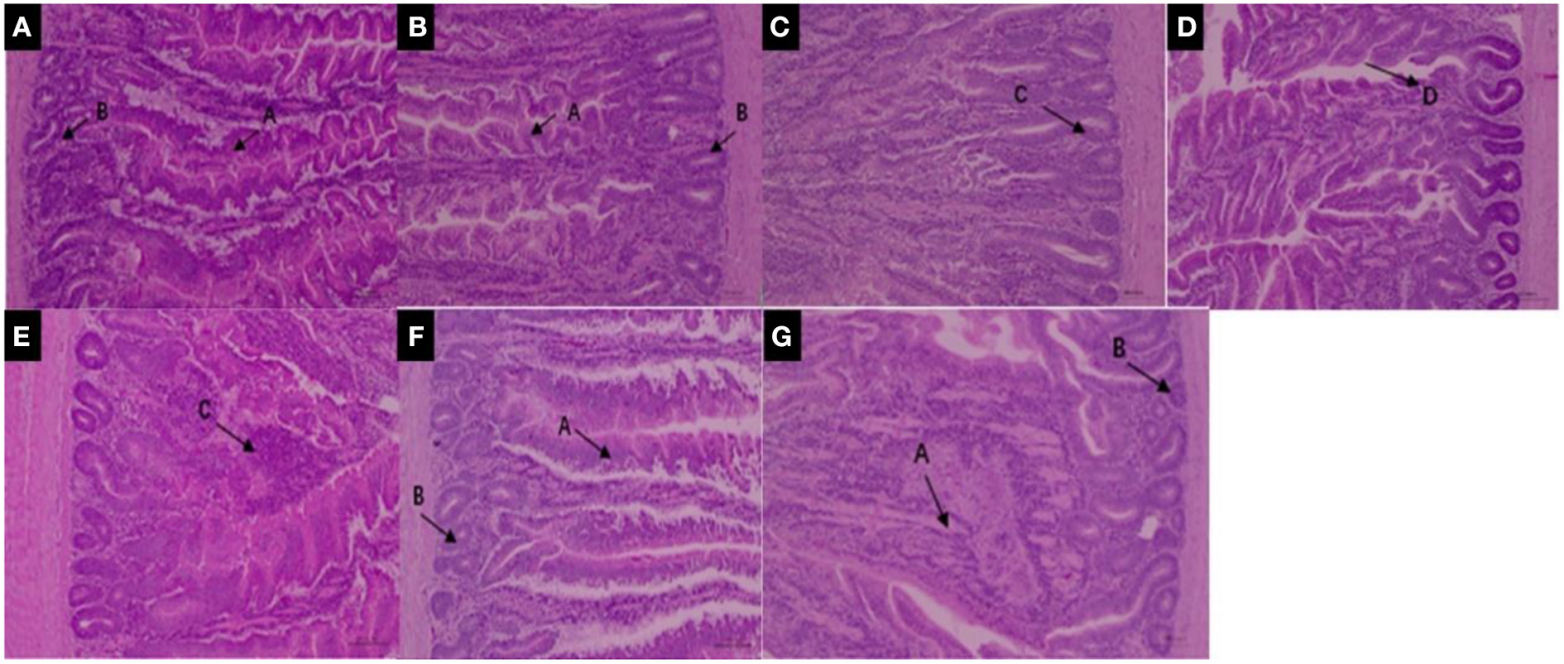
**(A–D)** Duodenum structure under 100× magnification during deltamethrin treatment for 0 **(A)**, the 1st **(B)**, 7th **(C)**, and 14th day **(D)**. **(E–G)** Duodenum structure under 100× magnification at the 1st **(E)**, 7th **(F)**, and 14th day **(G)** during deltamethrin treatment withdrawal. A, intestinal villi; B, intestinal glands (intestinal crypts); C, local mucosal lymphocyte infiltration; D, absence of intestinal villi epithelium.

**Figure 4:**
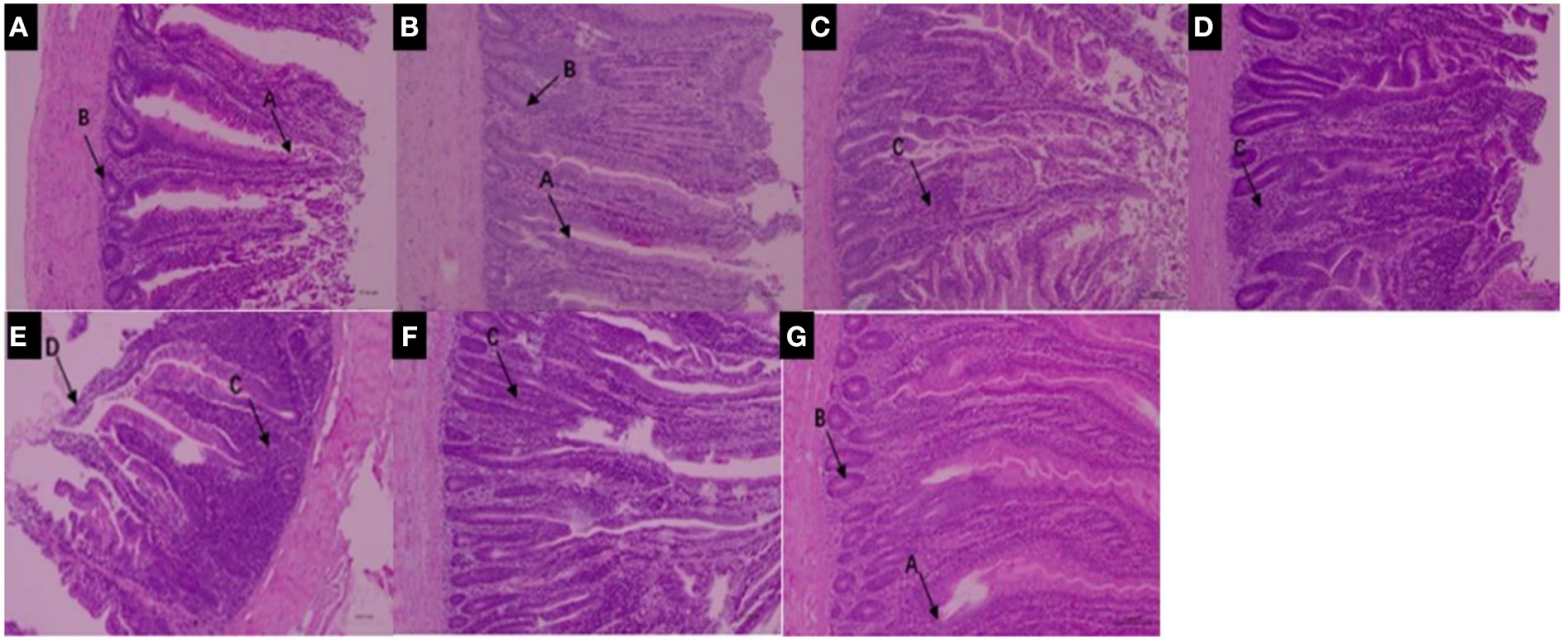
**(A–D)** Jejunum structure under 100× magnification during deltamethrin treatment for 0 **(A)**, the 1st **(B)**, 7th **(C)**, and 14th day **(D)**. **(E–G)** Jejunum structure under 100× magnification at the 1st **(E)**, 7th **(F)**, and 14th day **(G)** during deltamethrin treatment withdrawal. A, intestinal villi; B, intestinal glands (intestinal crypts); C, local mucosal lymphocyte infiltration; D, absence of intestinal villi epithelium.

**Figure 5:**
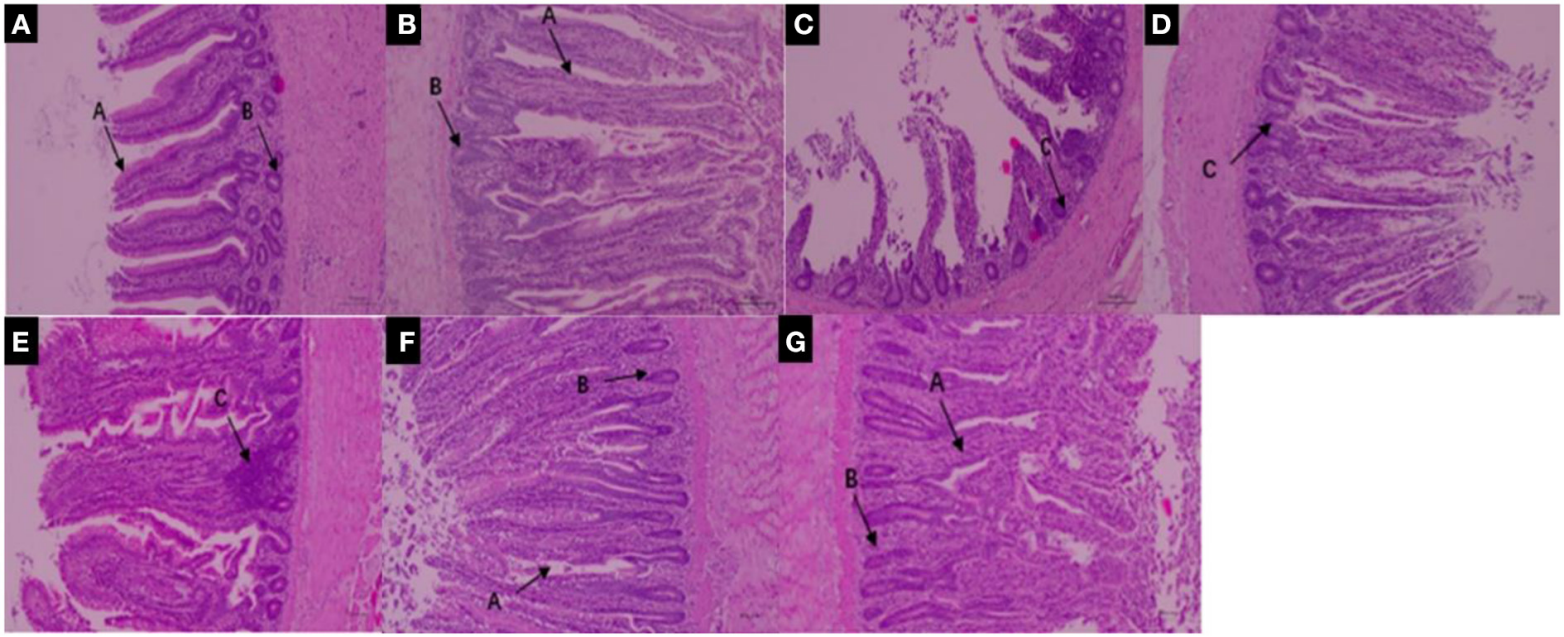
**(A–D)** Ileum structure under 100× magnification during treatment with deltamethrin for 0 **(A)**, the 1st **(B)**, 7th **(C)**, and 14th day **(D)**. **(E–G)** Ileum structure under 100× magnification at the 1st **(E)**, 7th **(F)**, and 14th day **(G)** during deltamethrin treatment withdrawal. A, intestinal villi; B, intestinal glands (intestinal crypts); C, local mucosal lymphocyte infiltration; D, absence of intestinal villi epithelium.

Many studies have found that pyrethroids have potential toxicity to humans and mammals (57–59). The effects of pyrethroids on the intestinal health of aquatic organisms also been reported (60, 61). Deltamethrin is distributed in different organs, and bioaccumulation is higher in the intestinal tract of aquatic organisms than in other organs (62). It has been reported that direct contact of toxicants with the intestinal tract affects intestinal bacteria hosts, through the regulation of a variety of cellular signaling pathways (63, 64). The structure of the small intestine can reflect physiological functions such as movement, digestion, and absorption. The present study therefore investigated the effects of deltamethrin on intestinal health in laying chicken. Increased deltamethrin treatment period was associated with gradual increases in injury to the duodenum, jejunum, and ileum tissues (Figures 3A–D, 4A–D, 5A–D). At the 7th day of deltamethrin treatment, numerous lymphoid follicles appeared at the bottom of local mucosa, and the intestinal villi were degenerated and necrotic (Figures 3C, 4C, 5C). At the 1st day during withdrawal, intestinal villi increased in number and were closely arranged, and the number of inflammatory cells decreased (Figures 3E–G, 4E–G, 5E–G). During withdrawal, the gradual repair of small intestinal mucosal cells and the strengthening of the defense functions might be associated with the decreased concentrations of deltamethrin, consistent with the results of previous studies (60, 65, 66).

Intestinal wall thickness, villus height, and crypt depth are important indexes to measure the digestibility and absorption capacity of the small intestine. In this study, intestinal wall thickness and duodenum height were not significantly altered by deltamethrin treatment (Table 3), consistent with the results of previous studies (67). During the deltamethrin treatment period, the crypt depth gradually increased compared with day zero of treatment, but there were no significant differences among the other time points. At 1st day of deltamethrin treatment, duodenum villus height and crypt depth were significantly greater than they were at the 1st and 7th day of withdrawal period (*p* < 0.05), but there was no significant difference among other time points (*p* > 0.05) (Table 3). In the jejunum, intestinal wall thickness, villus height, and crypt depth were higher during and after deltamethrin treatment compared with day zero of treatment. The intestinal wall thickness, villus height, and crypt depth were significantly greater at the 14th day of withdrawal than at day zero of deltamethrin treatment (*p* < 0.05), but the difference in villus height/crypt depth at the 14th day of withdrawal was not significant (*p* > 0.05) (Table 3). Ileum wall thickness gradually decreased during deltamethrin treatment, then continuously increased during the withdrawal period; the ileum wall thickness at the 14th day of during withdrawal period was significantly higher than it was at the day zero of deltamethrin treatment (*p* < 0.05). However, there was no significant difference among other time points (*p* > 0.05). In the ileum, there was no significant difference among different time points in villus height, which first increased, then decreased during deltamethrin treatment, and increased again during withdrawal period. Ileum crypt depth also continued to increase during withdrawal period, with significantly increased crypt depth at the 14th day of withdrawal compared with day zero of deltamethrin treatment (*p* < 0.05), although the differences were not significant at other time points (*p* > 0.05). The villus height and crypt depth of the ileum had significantly higher values at the 1st day of withdrawal than at the 14th day of treatment during deltamethrin treatment and withdrawal (Table 3). Thus, investigation of laying chicken small intestinal tissue morphology showed that the intestinal wall thickness was greater in each segment of the small intestine at the 14th day of deltamethrin withdrawal than at day zero of deltamethrin treatment. Deltamethrin had no significant effect on villus height in the duodenum and the ileum. In the jejunum, villus height significantly increased with daily deltamethrin administration. Deltamethrin treatment also significantly increased the crypt depth of each segment. Villus height/crypt depth are compre chickensive indicators of small intestine functional status. Decreased villus height/crypt depth ratios correspond to decreased digestive and absorptive functions, which often indicated a sign of diarrhea. These results showed deltamethrin treatment can negatively influence the intestinal health of laying chickens.

**Table 3:** Effects of deltamethrin on intestinal tissue morphology.

**Project**	**Administration of drugs day 0**	**Administration of drugs day 1**	**Administration of drugs day 7**	**Administration of drugs day 14**	**Withdrawal day 1**	**Withdrawal day 7**	**Withdrawal day 14**
**Intestinal wall thickness (μm)**							
Duodenum	1575.95 ± 193.79	1608.93 ± 128.66	1833.65 ± 188.59	1784.83 ± 196.86	1743.80 ± 289.65	1757.78 ± 312.10	1736.12 ± 218.32
Jejunum	926.80 ± 48.78^a^	1197.95 ± 159.84^ab^	1394.52 ± 282.89^bc^	1406.52 ± 129.53^bc^	1182.52 ± 159.66^ab^	1379.10 ± 312.33^bc^	1625.02 ± 302.83^c^
Ileum	890.50 ± 118.52^a^	1149.90 ± 159.78^ab^	1029.68 ± 295.58^ab^	928.47 ± 153.25^ab^	971.27 ± 231.15^ab^	1081.83 ± 167.03^ab^	1185.65 ± 208.52^b^
**Villus height (μm)**							
Duodenum	947.25 ± 73.50	1028.48 ± 145.60	1122.77 ± 149.26	1115.40 ± 173.48	973.72 ± 181.18	1013.63 ± 223.60	1050.87 ± 140.15
Jejunum	577.96 ± 69.16^a^	727.48 ± 111.56^ab^	835.50 ± 171.46^bc^	872.77 ± 112.35^bc^	726.65 ± 128.57^ab^	867.70 ± 181.94^bc^	1001.67 ± 233.97^c^
Ileum	444.05 ± 84.17	607.15 ± 130.58	552.68 ± 137.94	457.61 ± 121.52	550.90 ± 125.57	555.62 ± 67.12	622.15 ± 188.63
**Crypt depth (μm)**							
Duodenum	233.43 ± 53.06^ab^	230.70 ± 32.44^a^	312.07 ± 52.71^c^	280.32 ± 24.36^abc^	283.03 ± 44.89^abc^	299.03 ± 36.42^c^	286.42 ± 25.06^bc^
Jejunum	143.70 ± 23.01^a^	184.85 ± 34.86^ab^	247.08 ± 28.61^bc^	263.10 ± 45.61^c^	207.70 ± 36.37^bc^	237.85 ± 64.88^bc^	271.83 ± 65.66^c^
Ileum	117.88 ± 13.22^a^	152.98 ± 11.76^bc^	145.47 ± 26.13^abc^	172.13 ± 26.88^cd^	128.02 ± 20.87^ab^	168.67 ± 14.77^cd^	184.43 ± 22.49^d^
**Villus height/Crypt depth**							
Duodenum	4.17 ± 0.73^ab^	4.57 ± 1.12^b^	3.69 ± 0.83^ab^	4.00 ± 0.73^ab^	3.44 ± 0.31^a^	3.38 ± 0.55^a^	3.70 ± 0.66^ab^
Jejunum	4.11 ± 0.87	3.96 ± 0.15	3.37 ± 0.52	3.37 ± 0.53	3.54 ± 0.59	3.75 ± 0.72	3.88 ± 1.22
Ileum	3.78 ± 0.59^bc^	3.97 ± 0.79^bc^	3.81 ± 0.78^bc^	2.68 ± 0.75^a^	4.28 ± 0.53^c^	3.29 ± 0.19^ab^	3.35 ± 0.75^ab^

Superscript letters after mean values indicate statistically significant differences at p < 0.05.

## Conclusion

We here found that oral administration of deltamethrin had no significant effect on relative organ weight in laying chickens (*p* > 0.05). However, deltamethrin did have a great influence on blood biochemical indexes, which might be caused by liver injury or kidney inflammation. After deltamethrin enters the body of a laying chicken, it easily causes varying degrees of damage to the liver and intestinal tissue. During deltamethrin treatment withdrawal, intestinal repair capacity is stronger than the liver. Therefore, we conclude that deltamethrin exposure in laying chickens can induce changes in relative organ weight, alter blood biochemistry, and damage liver and intestinal tissues. Deltamethrin residues have extremely adverse effects on animal-derived food safety. Appropriate deltamethrin application in laying chickens ishould be carefully adhered to, to ensure the animal food safety and human health.

## Data availability statement

The original contributions presented in the study are included in the article/supplementary material, further inquiries can be directed to the corresponding authors.

## Ethics statement

The animal study was reviewed and approved by all experiments involving laying chickens were approved by the Animal Use and Care Committee of the Chinese Academy of Agricultural Sciences (Beijing, China, FRI-CAAS-20180527).

## Author contributions

YL, MH, XG, and CL contribute to revising it critically for important intellectual content and approved the version to be published. CL, YT, MJ, XC, HL, and YG have participated sufficiently in the work to take public responsibility for appropriate portions of the content and made a substantial contribution to the concept and design, acquisition of data or analysis, and interpretation of data. YL and MH co-completed the experimental technical route writing and finished the whole experiment work together. MH wrote the first draft of the manuscript. All authors contributed to the article and approved the submitted version.
